# Supplementary material for: Peer influence on West Point cadets’ Civil War allegiances
Source: Proc Natl Acad Sci U S A. 2026 May 27;123(22):e2529668123. doi: 10.1073/pnas.2529668123 (PMC13229209; doi:10.1073/pnas.2529668123)
Supplement: Supplementary file 1 — Appendix 01 (PDF) [file pnas.2529668123.sapp.pdf]

Supporting Information for

Peer Influence on West Point Cadets' Civil War Allegiances

Yuchen Guo, Matthew O. Jackson and Ruixue Jia

Matthew O. Jackson.
E-mail:jacksonm@stanford.edu

This PDF file includes:

Figs. S1 to S3
Tables S1 to S18
SI References

1. Data Construction and Description

A. Data Construction. We construct a dataset of West Point cadets from 1820 to 1860. We manually collect and organize information on graduates, including their Union or Confederate affiliation during the Civil War, class ranking at graduation, place of birth, year of birth, and military rank in 1865, from the *Biographical Register of the Officers and Graduates of the United States Military Academy* and other sources. We first present two examples of coding from main source in Figure S1.

Civil War Affiliation We determine military allegiance based on whether the individual served with the Union or the Confederacy during the Civil War. This is exemplified by statements such as "He took part in the Rebellion of 1861-66 against the United States" or "He served in the Rebellion of the Seceding States."

For those whose biographies do not clearly specify a side, we deduce their affiliation from the forces they served with during the war, including volunteer armies and local militias. For instance, service in the New York State Military during the Civil War categorizes them as Union members.

We cross reference our data with additional sources including *Find a Grave*, Wikipedia, *Rebels from West Point*, and *Southern Historical Society Papers*.

Class Rank at Graduation Since the number of cadets graduating in each West Point cohort varied, direct comparisons of class ranks across different cohorts are not meaningful. To ensure comparability, we classify cadets into percentiles based on their graduation ranking. The top 10% were assigned a score of 100, the bottom 10% a score of 10, and so on.

Year of Birth In a few cases where an individual's year of birth was unavailable in the *Biographical Register* , we supplement this information by using details from *Find a Grave* and *FamilySearch*.

Birth Place The birthplace information reported in the *Biographical Register of the Officers and Graduates of the United States Military Academy* is occasionally incomplete or imprecise, particularly with respect to county-level identifiers. To address these limitations, we supplement the original records using genealogical sources, including *Find a Grave* and *FamilySearch*, which provide more detailed and consistent location information. These sources allow us to recover missing county-level data and to correct ambiguous or inaccurate birthplace entries, thereby improving the geographic precision of our measures.

Military Rank in 1865 For cadets who joined the Union, we determine their official military rank in 1865 from personal biographies. For cadets who joined the Confederacy, we refer to sources including *Rebels from West Point*, *Southern Historical Society Papers*, *Confederate Military History*, and Wikipedia, for military rank descriptions.

If the personal information did not include a specific military rank in 1865, we approximated it based on the individual's military career trajectory. For example, if an individual's career record states: {'1846: Second Lieutenant', '1864: Major', '1870: Colonel'}, we assign the rank held closest to 1865, but no later than 1865. In this case, the individual's rank in 1865 would be Major. Since every change in a person's military rank is recorded, if there is no mention of a change, we assume the rank remained the same. We focus on formal military ranks rather than honorary ranks and volunteer military ranks. Military ranks are grouped into 11 levels, with General ranked as 11 and Third Lieutenant ranked as 1.

Participation in the Mexican-American War The information is constructed from the *Biographical Register of the Officers and Graduates of the United States Military Academy*. We record individuals who directly participated in the Mexican-American War, including those engaged in combat or other military operations.

Genealogical and Census Data Linkage We supplemented the West Point biographical records with individual- and family-level information via manual linkage to genealogical (*FamilySearch*) and US Census databases (*Ancestry*). Using cadets' names and birth/death dates, we first searched *FamilySearch* to identify the correct individual, recorded the unique page identifier, and collected birthplace, birthdate, residence around 1860, basic marital information and parental information. We then searched *Ancestry* for the 1860 Census and confirmed matches based on consistency in name, age, birthplace, and residence, extracting occupation, residence, and reported real and personal estate values. Finally, we consulted the 1860 Slave Schedules on *Ancestry*, using name and residence consistency to identify slaveholding status. Using cadets' county of residence, we further linked each individual to county-level characteristics in 1860, including the number of churches[*] and the voting electoral returns[†].

[*] Inter-university Consortium for Political and Social Research. United States Historical Election Returns, 1824-1968.

[†] Haines, Michael R., and Inter-university Consortium for Political and Social Research. Historical, Demographic, Economic, and Social Data: The United States, 1790-2002.

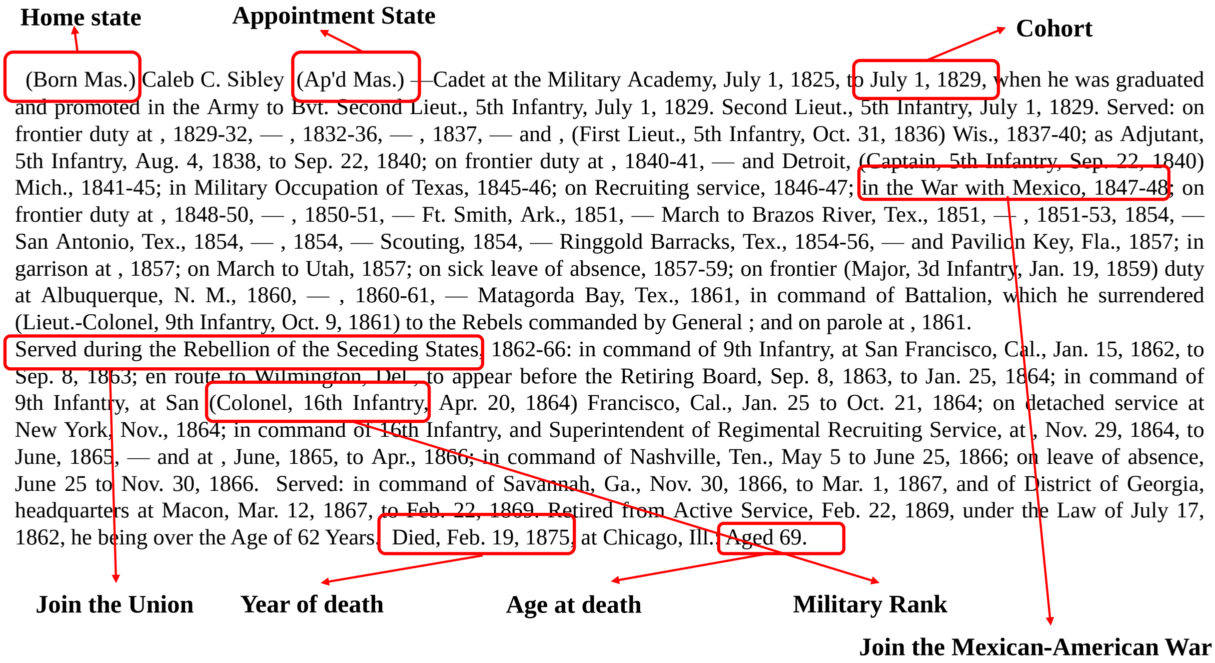

A: Example of coding the the Union

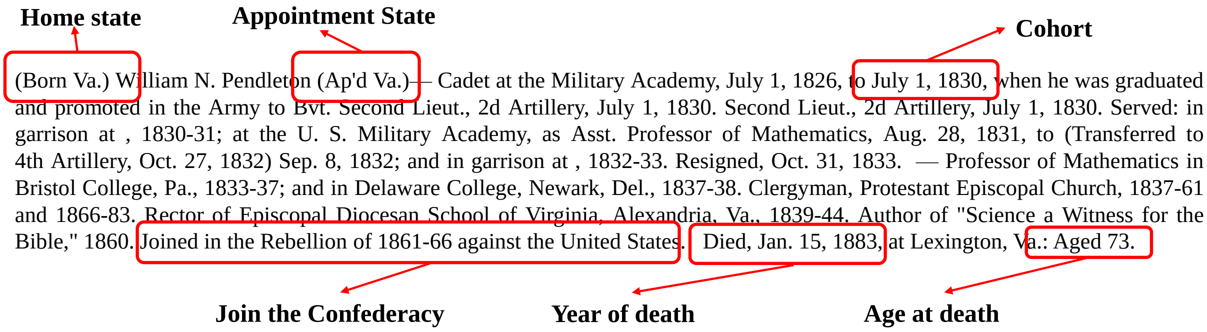

B: Example of coding the Confederacy

Fig. S1. Text examples

Note. This figure presents text examples from Cullum (1). We use these texts as our main data source and complement the data with information from additional sources

B. Classification of States.

Table S1. Classification of states

	Slave share (%)	1% threshold	5% threshold	10% threshold	4 groups
South Carolina	57.2	Slave-State	>5%	>10%	Heavy-Slave State
Mississippi	55.2	Slave-State	>5%	>10%	Heavy-Slave State
Louisiana	46.9	Slave-State	>5%	>10%	Heavy-Slave State
Alabama	45.1	Slave-State	>5%	>10%	Heavy-Slave State
Florida	44.0	Slave-State	>5%	>10%	Heavy-Slave State
Georgia	43.7	Slave-State	>5%	>10%	Heavy-Slave State
North Carolina	33.7	Slave-State	>5%	>10%	Heavy-Slave State
Virginia	30.7	Slave-State	>5%	>10%	Mid-Slave State
Texas	30.2	Slave-State	>5%	>10%	Mid-Slave State
Arkansas	25.5	Slave-State	>5%	>10%	Mid-Slave State
Tennessee	24.8	Slave-State	>5%	>10%	Mid-Slave State
Kentucky	19.5	Slave-State	>5%	>10%	Border-State
Maryland	12.7	Slave-State	>5%	>10%	Border-State
Missouri	9.7	Slave-State	>5%	<10%	Border-State
District of Columbia	4.4	Slave-State	<5%	<10%	Border-State
Delaware	1.6	Slave-State	<5%	<10%	Border-State
New Jersey	0.01	Free-State	<5%	<10%	Free-State
New York	0.0	Free-State	<5%	<10%	Free-State
Pennsylvania	0.0	Free-State	<5%	<10%	Free-State
Ohio	0.0	Free-State	<5%	<10%	Free-State
Illinois	0.0	Free-State	<5%	<10%	Free-State
Indiana	0.0	Free-State	<5%	<10%	Free-State
Massachusetts	0.0	Free-State	<5%	<10%	Free-State
Wisconsin	0.0	Free-State	<5%	<10%	Free-State
Michigan	0.0	Free-State	<5%	<10%	Free-State
Iowa	0.0	Free-State	<5%	<10%	Free-State
Maine	0.0	Free-State	<5%	<10%	Free-State
Connecticut	0.0	Free-State	<5%	<10%	Free-State
California	0.0	Free-State	<5%	<10%	Free-State
New Hampshire	0.0	Free-State	<5%	<10%	Free-State
Vermont	0.0	Free-State	<5%	<10%	Free-State
Rhode Island	0.0	Free-State	<5%	<10%	Free-State
Minnesota	0.0	Free-State	<5%	<10%	Free-State
Oregon	0.0	Free-State	<5%	<10%	Free-State

Note. This table presents different ways we employ to classify home states of West Point cadets.

C. Summary Statistics.

Table S2. Summary statistics

	Mean	Std. Dev.	Min.	Max.	Obs.
Panel A: Heavy-Slave States (slave share $>$ 33%)					
Joining the Union (War Participants)	0.107	0.310	0	1	122
Joining the Union (All)	0.062	0.242	0	1	210
Joining the War	0.581	0.495	0	1	210
Class Rank	53.810	30.068	10	100	210
Age in 1860	40.530	11.315	23	65	210
Slave Pop. Share (state level)	45.370	9.467	33.4	57.2	210
Cohort	1841.290	11.716	1820	1860	210
Join Mex.-Am. War	0.276	0.448	0	1	210
Cadet Slave Ownership	0.352	0.480	0	1	122
Slave Pop. Share (County-level)	49.415	16.553	13.191	84.999	108
Free-State Father	0.204	0.405	0	1	103
Free-State Mother	0.080	0.273	0	1	100
Free-State Wife	0.172	0.379	0	1	122
Panel B: Border and Mid-Slave States (1%$<$slave share $\leq$ 33%)					
Joining the Union (War Participants)	0.477	0.500	0	1	266
Joining the Union (All)	0.276	0.448	0	1	460
Joining the War	0.578	0.494	0	1	460
Class Rank	54.326	28.288	10	100	460
Age in 1860	41.841	10.461	22	62	460
Slave Pop. Share	20.739	9.379	1.6	30.700	460
Cohort	1839.761	10.890	1820	1860	460
Join Mex.-Am. War	0.317	0.466	0	1	460
Slave Ownership	0.207	0.406	0	1	266
Slave Pop. Share (County-level)	24.269	18.718	0.446	71.269	244
Free-State Father	0.132	0.340	0	1	234
Free-State Mother	0.137	0.345	0	1	233
Free-State Wife	0.297	0.458	0	1	266
Panel C: Free States (slave share $<$ 1%)					
Joining the Union (War Participants)	0.919	0.274	0	1	540
Joining the Union (All)	0.512	0.500	0	1	968
Joining the War	0.558	0.497	0	1	968
Class Rank	57.066	28.663	10	100	968
Age in 1860	41.535	10.899	21	63	968
Slave Pop. Share	0.000	0.002	0	0.010	968
Cohort	1840.346	11.337	1820	1860	968
Join Mex.-Am. War	0.305	0.461	0	1	968
Cadet Slave Ownership	0	0	0	0	540
Slave Pop. Share (County-level)	0.000	0.000	0	0.012	433
Free-State Father	0.868	0.338	0	1	471
Free-State Mother	0.890	0.314	0	1	462
Free-State Wife	0.467	0.499	0	1	540

Note. This table presents summary statistics for the key variables used in our analysis. *Slave Pop. Share (county level)* and *Slave Pop. Share (state level)* are expressed in percentages. Variables *Free-State Father*, *Free-State Mother*, *Free-State Wife*, and *Cadet Slave Ownership* are collected only for individuals who participated in the Civil War.

Table S3. Summary statistics: Cohort-level

	Mean	Std. Dev.	Min.	Max.	Obs.
Panel A: Heavy-Slave States (slave share $>$ 33%)					
Number of Graduates	5.122	2.532	0	11	41
Fraction of Graduates	0.127	0.057	0	0.263	41
Panel B: Border and Mid-Slave States (1%$<$slave share $\leq$ 33%)					
Number of Graduates	11.220	3.863	2	19	41
Fraction of Graduates	0.280	0.080	0.059	0.480	41
Panel C: Free States (slave share $<$ 1%)					
Number of Graduates	23.610	5.572	12	33	41
Fraction of Graduates	0.593	0.085	0.462	0.824	41

Note. This table presents summary statistics at the cohort level.

D. Correlations between Slave Share and Allegiances.

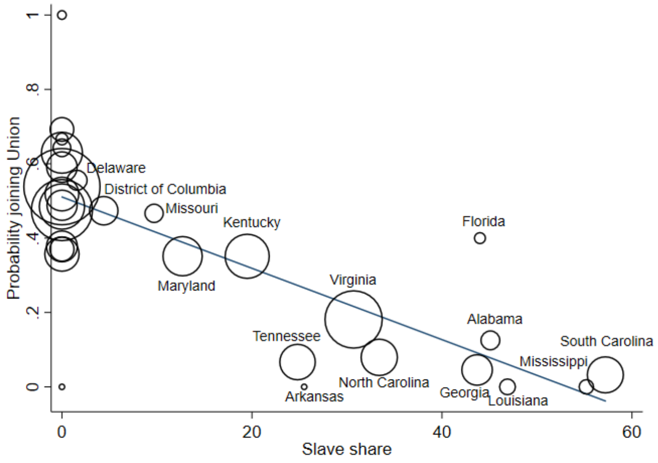

A: Probability of joining the Union

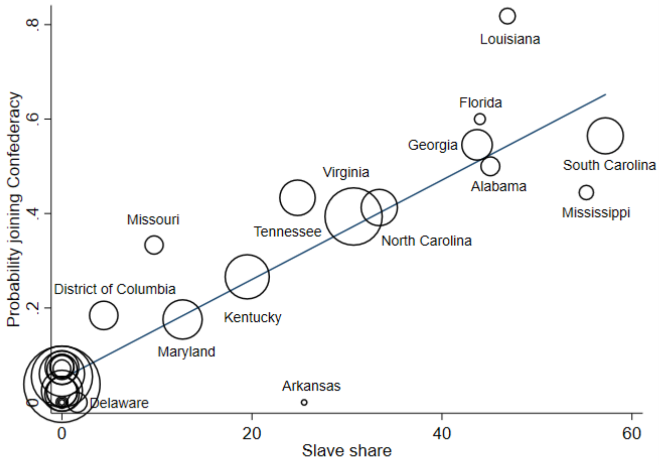

B: Probability of joining the Confederacy

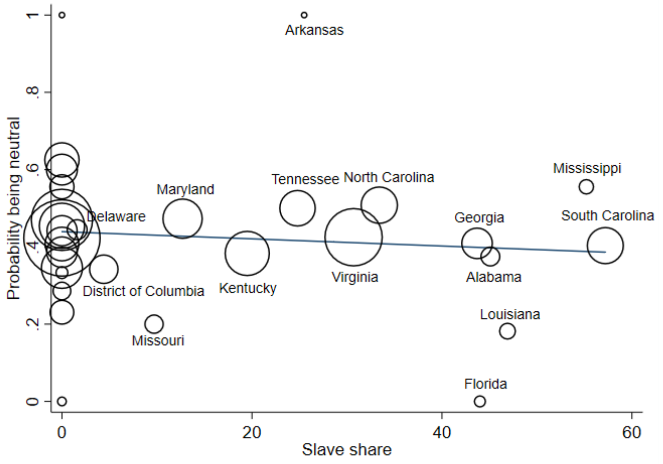

C: Probability of being neutral

Fig. S2. Slave share and cadet's choices

Note. The sample for this analysis consists of all cadets, including those who did not participate in the war. This figure illustrates the correlations between the proportion of slaves in a state and the likelihood of a cadet from that state opting to join the Union army (A), the Confederacy (B), or abstain from participating (C).

E. Peers and Personal Characteristics.

Table S4. Correlations between peers and personal characteristics

Dependent var (sd)	Share Free-State peers:1820-1860							
	War Participants				All			
	Slave States		Free States		Slave States		Free States	
	(1)	(2)	(3)	(4)	(5)	(6)	(7)	(8)
Age in 1860	-0.004	0.012	-0.004	-0.003	-0.007	0.012	0.005	-0.003
	(0.031)	(0.037)	(0.027)	(0.028)	(0.026)	(0.037)	(0.019)	(0.028)
Class Rank	-0.002	-0.002	0.001	0.001	-0.001	-0.002	0.000	0.001
	(0.002)	(0.002)	(0.001)	(0.002)	(0.001)	(0.002)	(0.001)	(0.002)
Slave Pop. Share (sd)	-0.107**	-0.069	-0.001	0.011	-0.030	-0.069	-0.035	0.011
	(0.049)	(0.063)	(0.042)	(0.040)	(0.038)	(0.063)	(0.030)	(0.040)
Cohort (t)	-0.002	0.017	0.007	0.009	-0.009	0.017	0.010	0.009
	(0.030)	(0.036)	(0.027)	(0.028)	(0.025)	(0.036)	(0.018)	(0.028)
Free-State Father		-0.005		0.013		-0.005		0.013
		(0.186)		(0.156)		(0.186)		(0.156)
Free-State Mother		0.347		-0.124		0.347		-0.124
		(0.216)		(0.182)		(0.216)		(0.182)
Free-State Wife		-0.073		0.033		-0.073		0.033
		(0.141)		(0.101)		(0.141)		(0.101)
Joint test p-value	0.190	0.336	0.130	0.370	0.739	0.336	0.212	0.370
Observations	388	324	540	456	670	324	968	456
R-squared	0.015	0.027	0.013	0.017	0.003	0.027	0.006	0.017

Note. This table presents the correlations between peer composition and individual traits. The joint test on overall correlations are statistically undistinguished from zero. The standard errors presented in the parentheses are obtained through bootstrapping with 400 resampling iterations. ***$p < 0.01$, **$p < 0.05$, *$p < 0.1$.

2. Additional Results

A. Alternative Thresholds.

Table S5. Peer composition and allegiance choice: Different thresholds

Dependent var	Join the Union: War Participants							
	Thresholds: 5%				Thresholds: 10%			
	Slave States		Free States		Slave States		Free States	
	(1)	(2)	(3)	(4)	(5)	(6)	(7)	(8)
Share Free-State Peers (sd)	0.061***	0.065***	-0.008	-0.011	0.064***	0.064***	-0.007	-0.009
	(0.022)	(0.022)	(0.012)	(0.012)	(0.024)	(0.024)	(0.013)	(0.013)
Age in 1860	-0.008	-0.003	0.004	0.003	-0.014	-0.009	0.006	0.005
	(0.015)	(0.016)	(0.007)	(0.008)	(0.016)	(0.016)	(0.007)	(0.007)
Class Rank	0.001	0.001	-0.001**	-0.001**	0.001	0.001	-0.001**	-0.001**
	(0.001)	(0.001)	(0.000)	(0.000)	(0.001)	(0.001)	(0.000)	(0.000)
Slave Pop. Share (sd)	-0.182***		-0.033*		-0.177***		-0.060***	
	(0.021)		(0.018)		(0.021)		(0.020)	
Cohort	-0.009	-0.004	0.007	0.006	-0.015	-0.009	0.008	0.007
	(0.015)	(0.015)	(0.007)	(0.007)	(0.015)	(0.016)	(0.007)	(0.007)
State FEs	N	Y	N	Y	N	Y	N	Y
Dependent var. mean	0.317	0.317	0.911	0.911	0.308	0.308	0.905	0.905
Observations	353	353	575	575	341	341	587	587
R-squared	0.179	0.238	0.034	0.052	0.178	0.238	0.055	0.072

Note. The standard errors reported in the parentheses are derived from bootstrapping with 400 resampling iterations. ***$p < 0.01$, **$p < 0.05$, *$p < 0.1$.

B. Cohort-Clustered and Bootstrap Cohort-Clustered SE.

Table S6. Peer composition and allegiance choice: Variations on standard errors

Dependent var	Join the Union: War Participants							
	Slave States				Free States			
	(1)	(2)	(3)	(4)	(5)	(6)	(7)	(8)
A. Cohort level bootstrap								
Share Free-State Peers (sd)	0.080***	0.058***	0.054***	0.056***	-0.003	-0.005	-0.006	-0.005
	(0.020)	(0.014)	(0.014)	(0.017)	(0.015)	(0.015)	(0.015)	(0.015)
Controls	N	Y	Y	Y	N	Y	Y	Y
State FEs	N	N	Y	Y	N	N	Y	Y
Dependent var. mean	0.361	0.361	0.361	0.355	0.919	0.919	0.919	0.930
ICC	0.000	0.000	0.000	0.000	0.028	0.016	0.015	0.025
Observations	388	388	388	324	540	540	540	456
R-squared	0.028	0.234	0.287	0.363	0.000	0.017	0.030	0.067
B. Cohort level cluster								
Share Free-State Peers (sd)	0.080***	0.058***	0.054***	0.056***	-0.003	-0.005	-0.006	-0.005
	(0.020)	(0.013)	(0.019)	(0.020)	(0.012)	(0.012)	(0.013)	(0.012)
Controls	N	Y	Y	Y	N	Y	Y	Y
State FEs	N	N	Y	Y	N	N	Y	Y
Dependent var. mean	0.361	0.361	0.361	0.355	0.919	0.919	0.919	0.930
ICC	0.000	0.000	0.000	0.000	0.028	0.016	0.015	0.025
Observations	388	388	388	324	540	540	540	456
R-squared	0.028	0.234	0.287	0.363	0.000	0.017	0.030	0.067

Note. Panel A reports the corresponding re-estimates using bootstrapped standard errors clustered at the cohort level with 400 resampling iterations. Panel B reports the re-estimation of the baseline results using robust standard errors clustered at the cohort level. (This table presents the impact of the fraction of peers from Free States in a cadet's cohort on that cadet's decision to join the Union. The control variables consist of *Age in 1860*, *Class Rank*, state level *Slave Population Share (sd)*, and *Cohort*. In addition, columns (4) and (8) further control for cadet slave ownership as well as family background characteristics, including whether the cadet's father, mother, or wife was from a free state. Columns (1)-(4) focus on cadets from Slave States and Columns (5)-(8) on those from Free States. ***$p < 0.01$, **$p < 0.05$, *$p < 0.1$.)

C. Different Age Groups.

Table S7. Peer composition and allegiance choice: Variations in age groups

Dependent var	Join the Union: War Participants					
	Slave States			Free States		
	(1)	(2)	(3)	(4)	(5)	(6)
A. $Age < 60$						
Share Free-State Peers (sd)	0.074***	0.052**	0.048**	-0.002	-0.004	-0.005
	(0.024)	(0.021)	(0.022)	(0.013)	(0.012)	(0.012)
Controls	N	Y	Y	N	Y	Y
State FEs	N	N	Y	N	N	Y
Dependent var. mean	0.359	0.359	0.359	0.920	0.920	0.920
Observations	384	384	384	535	535	535
R-squared	0.024	0.236	0.288	0.000	0.014	0.027
B. $Age < 50$						
Share Free-State Peers (sd)	0.080***	0.059***	0.053**	-0.005	-0.008	-0.009
	(0.026)	(0.021)	(0.022)	(0.013)	(0.013)	(0.013)
Controls	N	Y	Y	N	Y	Y
State FEs	N	N	Y	N	N	Y
Dependent var. mean	0.353	0.353	0.353	0.921	0.921	0.921
Observations	334	334	334	471	471	471
R-squared	0.028	0.248	0.301	0.000	0.025	0.041

Note. Panel A restricts the sample to cadets younger than 60 at the time of Civil War participation. Panel B further restricts the sample to those younger than 50. Columns (1)-(3) focus on cadets from Slave States and Columns (4)-(6) on those from Free States. ***$p < 0.01$, **$p < 0.05$, *$p < 0.1$.)

D. Home State Economic Proxies and War Allegiances.

Table S8. Home state economic proxies and war allegiance

Dependent var	Join the Union: War Participants							
	Slave States				Free States			
	(1)	(2)	(3)	(4)	(5)	(6)	(7)	(8)
Share Free-State Peers (sd)	0.058***	0.058***	0.054***	0.053***	-0.005	-0.006	-0.005	-0.005
	(0.020)	(0.020)	(0.020)	(0.020)	(0.013)	(0.013)	(0.013)	(0.013)
Age in 1860	-0.012	-0.012	-0.005	-0.005	0.006	0.006	0.006	0.006
	(0.015)	(0.015)	(0.015)	(0.015)	(0.007)	(0.007)	(0.007)	(0.007)
Class Rank	0.001	0.001	0.000	0.000	-0.001**	-0.001*	-0.001*	-0.001*
	(0.001)	(0.001)	(0.001)	(0.001)	(0.000)	(0.000)	(0.000)	(0.000)
Cohort	-0.013	-0.013	-0.005	-0.005	0.008	0.008	0.008	0.008
	(0.015)	(0.015)	(0.015)	(0.015)	(0.007)	(0.007)	(0.007)	(0.007)
Slave Pop. Share (sd)	-0.216***	-0.217***	-0.102**	-0.109***	-0.007	-0.011	-0.013	-0.012
	(0.019)	(0.019)	(0.042)	(0.042)	(0.016)	(0.016)	(0.016)	(0.017)
ln Farmland Value per Capita		0.011	0.018	0.025		0.052	0.072	0.069
		(0.048)	(0.048)	(0.048)		(0.040)	(0.054)	(0.063)
ln Manufactured Product per Capita			0.233***	0.168			0.019	0.022
			(0.077)	(0.123)			(0.031)	(0.055)
Manufacturing Employment Share (sd)				0.035				-0.002
				(0.056)				(0.034)
Dependent var. mean	0.361	0.361	0.361	0.361	0.919	0.919	0.919	0.919
Observations	388	388	388	388	540	540	540	540
R-squared	0.234	0.234	0.252	0.253	0.017	0.020	0.021	0.021

Note. This table shows the association between war allegiances and several home state economic variables. The standard errors reported in the parentheses are derived from bootstrapping with 400 resampling iterations. $^{***}p < 0.01$, $^{**}p < 0.05$, $^{*}p < 0.1$.

E. Considering Religion and Voting.

As a proxy for underlying religious attitudes, we follow Fogel (2) by classifying denominations into two broad groups: evangelical churches (Congregationalists, Methodists and Presbyterians) and liturgical churches (Catholics, Lutherans, Episcopalians) plus Quakers; with the former group tending to be associated with pro-slavery constituencies, and the latter tending to be associated with anti-slavery constituencies in 1860, but with some being divided and/or not taking open stances. We use the number of churches of the given group per 1000 population as the measure. Although this categorization of religion captures some basic tendencies to support or oppose slavery across the whole United States at the time, churches in slave states (other than the small set of Quakers) tended to support or be ambiguous on slavery regardless of category, which may be why we find no effects of religious categories in the table below.

As expected, Republican vote share is positively associated with the probability of joining the Union. However, controlling for voting and its interaction with peer influence does not alter our main findings on slave population share or its interaction with peer effects, suggesting that slave share is a more powerful proxy for cadets' political-economic background.

Table S9. Peer composition and allegiance choice: Religion and voting

Dependent var	Join the Union: War Participants									
	Slave States					Border States				
	(1)	(2)	(3)	(4)	(5)	(6)	(7)	(8)	(9)	(10)
Share Free-State Peers (sd)	0.053**	0.053**	0.054**	0.056**	0.054**	0.068***	0.067***	0.068***	0.073**	0.074**
	(0.021)	(0.021)	(0.021)	(0.022)	(0.022)	(0.025)	(0.025)	(0.025)	(0.029)	(0.029)
Slave Share (County) × Share Free-State Peers (sd)	-0.036*	-0.040**	-0.043**	-0.047**	-0.041**	-0.027	-0.025	-0.027	-0.046*	-0.037
	(0.018)	(0.020)	(0.020)	(0.021)	(0.020)	(0.023)	(0.025)	(0.025)	(0.026)	(0.023)
Slave Share (County)	-0.166***	-0.170***	-0.165***	-0.142***	-0.161***	-0.187***	-0.187***	-0.184***	-0.163***	-0.183***
	(0.033)	(0.033)	(0.034)	(0.034)	(0.034)	(0.036)	(0.036)	(0.036)	(0.036)	(0.034)
Evangelical × Share Free-State Peers (sd)		-0.012					0.011			
		(0.018)					(0.023)			
Evangelical		-0.022					0.003			
		(0.026)					(0.033)			
Liturgical × Share Free-State Peers (sd)			-0.021					0.000		
			(0.024)					(0.026)		
Liturgical + Quaker			0.015					0.014		
			(0.036)					(0.045)		
Republican × Share Free-State Peers (sd)				-0.013					-0.017	
				(0.018)					(0.022)	
Republican				0.091**					0.090*	
				(0.043)					(0.053)	
Southern Democrat × Share Free-State Peers (sd)					-0.003					0.003
					(0.023)					(0.029)
Southern Democrat					-0.021					-0.020
					(0.045)					(0.046)
Controls	Y	Y	Y	Y	Y	Y	Y	Y	Y	Y
State FEs	Y	Y	Y	Y	Y	Y	Y	Y	Y	Y
Dependent var. mean	0.358	0.358	0.358	0.330	0.330	0.471	0.471	0.471	0.443	0.443
Observations	352	352	352	327	327	244	244	244	219	219
R-squared	0.341	0.343	0.343	0.349	0.337	0.295	0.295	0.295	0.318	0.308

Note. Our sample for this table is restricted to cadets from Slave States. For religion, we use the number of churches per 1,000 population as a proxy for a religious environment. The control variables consist of *Age in 1860*, *Class Rank*, and *Cohort*. Columns (4)–(5) and (9)–(10) have smaller sample sizes because Washington, DC did not have complete voting records prior to 1861. Columns (1)-(5) focus on cadets from Slave States and Columns (6)-(10) on those from Border States. The standard errors reported in the parentheses are derived from bootstrapping with 400 resampling iterations. ***$p < 0.01$, **$p < 0.05$, *$p < 0.1$.

F. Peer Influence by Cohort.

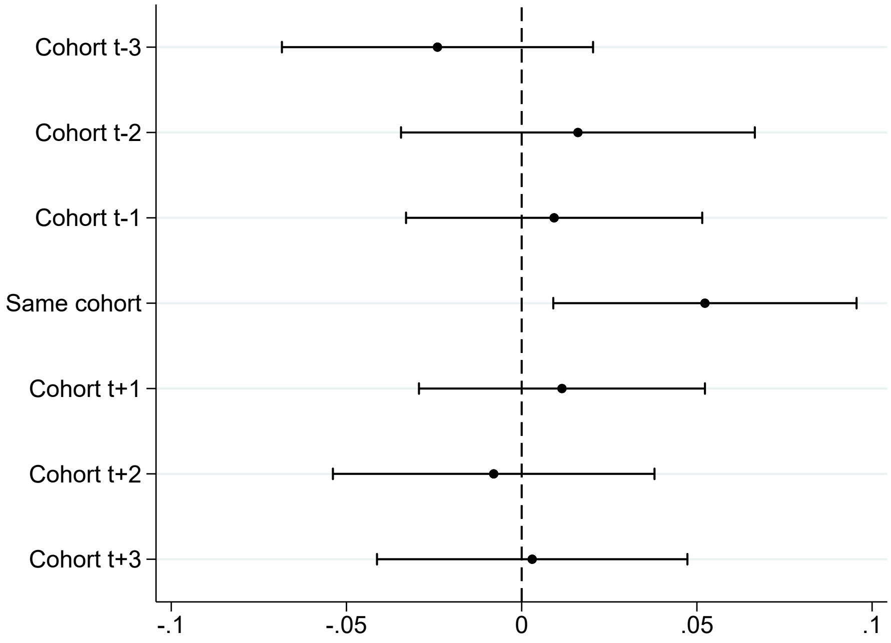

Fig. S3. Peer influence by cohort

Note. The sample for this analysis consists of the war-participating cadets from Slave States (i.e., with a slave population higher than 1%). This figure reports the coefficients of peers from the same cohort t and those from three cohorts before and after t. The coefficients are derived from a multiple regression that includes all controls from our baseline analysis. The bars represent 90% confidence intervals.

G. Peer Influence and Continuous Military Service Experience.

Table S10. Peer composition and allegiance choice: Continuous military service experience

Dependent var	Join the Union: War Participants					
	Non-continuous Military Service			Continuous Military Service		
	(1)	(2)	(3)	(4)	(5)	(6)
Share Free-State Peers (sd)	0.014	0.022	0.005	0.098***	0.063**	0.062**
	(0.039)	(0.035)	(0.038)	(0.029)	(0.026)	(0.027)
Age in 1860		-0.006	0.013		-0.017	-0.014
		(0.022)	(0.024)		(0.017)	(0.018)
Class Rank		-0.001	-0.001		0.002*	0.002*
		(0.001)	(0.001)		(0.001)	(0.001)
Slave Pop. Share (sd)		-0.171***			-0.218***	
		(0.030)			(0.024)	
Cohort		-0.011	0.006		-0.023	-0.020
		(0.021)	(0.022)		(0.016)	(0.017)
State FEs	N	N	Y	N	N	Y
Dependent var. mean	0.202	0.202	0.202	0.431	0.431	0.431
Observations	119	119	119	269	269	269
R-squared	0.001	0.230	0.347	0.039	0.272	0.323

Note. The sample for this analysis consists of all war-participating cadets. Columns (1)-(3) use the sample of cadets who remained out of military service for more than one year before the war, while Columns (4)-(6) use the sample of cadets who remained continuously in military service. The standard errors reported in the parentheses are derived from bootstrapping with 400 resampling iterations $^{***}p < 0.01$, $^{**}p < 0.05$, $^{*}p < 0.1$.

H. Peer Influence pre and post 1850.

Table S11. Peer composition and allegiance choice before and after 1850

Dependent var	Join the Union: War Participants			
	1820-1849		1850-1860	
	(1)	(2)	(3)	(4)
Share Free-State Peers (sd)	0.054**	0.045*	0.057*	0.062*
	(0.026)	(0.026)	(0.032)	(0.034)
Age in 1860	-0.001	0.018	-0.026	-0.030
	(0.020)	(0.020)	(0.022)	(0.026)
Class Rank	0.000	0.001	0.001	0.000
	(0.001)	(0.001)	(0.001)	(0.001)
Slave Pop. Share (sd)	-0.224***		-0.206***	
	(0.023)		(0.032)	
Cohort	-0.002	0.015	-0.013	-0.017
	(0.020)	(0.020)	(0.022)	(0.026)
State FEs	N	Y	N	Y
Dependent var.mean	0.389	0.389	0.318	0.318
Observations	234	234	154	154
R-squared	0.236	0.301	0.231	0.332

Note. The sample for this analysis consists of the war-participating cadets from Slave States (i.e., with a slave population higher than 1%). The standard errors reported in the parentheses are derived from bootstrapping with 400 resampling iterations. ***$p < 0.01$, **$p < 0.05$, *$p < 0.1$.

I. Controlling for Five-Year Fixed Effects.

Table S12. Peer composition and allegiance choice with five-year fixed effects

Dependent var	Join the Union: War Participants					
	Slave States			Free States		
	(1)	(2)	(3)	(4)	(5)	(6)
Share Free-State Peers (sd)	0.076***	0.062***	0.058**	-0.008	-0.009	-0.011
	(0.025)	(0.022)	(0.022)	(0.013)	(0.012)	(0.013)
Controls	N	Y	Y	N	Y	Y
State FEs	N	N	Y	N	N	Y
Five-Year Interval FEs	Y	Y	Y	Y	Y	Y
Dependent var. mean	0.361	0.361	0.361	0.919	0.919	0.919
Observations	388	388	388	540	540	540
R-squared	0.041	0.240	0.292	0.033	0.043	0.057

Note. This table presents the impact of the fraction of peers from Free States in a cadet's cohort on that cadet's decision to join the Union. We include fixed effects at five year intervals to allow for a shift in the environment. The control variables consist of *Age in 1860*, *Class Rank*, state level *Slave Population Share (sd)*, and *Cohort*. Columns (1)-(3) focus on cadets from Slave States and Columns (4)-(6) on those from Free States. The standard errors presented in the parentheses are obtained through bootstrapping with 400 resampling iterations. ***$p < 0.01$, **$p < 0.05$, *$p < 0.1$.

J. Graduate Peers vs. Dropout Peers.

Table S13. Considering dropout peers

Dependent var	Join the Union: War Participants			
	Slave States			
	(1)	(2)	(3)	(4)
Share Free-State Total Peers (sd)	0.058**	0.055**		
	(0.023)	(0.023)		
Share Free-State Graduates (sd)			0.059***	0.053**
			(0.022)	(0.022)
Share Free-State Dropouts (sd)			0.009	0.010
			(0.021)	(0.020)
Age in 1860	-0.011	-0.004	-0.012	-0.005
	(0.015)	(0.015)	(0.015)	(0.016)
Class Rank	0.001	0.001	0.000	0.001
	(0.001)	(0.001)	(0.001)	(0.001)
Slave Pop. Share (sd)	-0.216***		-0.217***	
	(0.019)		(0.019)	
Cohort	-0.014	-0.006	-0.015	-0.007
	(0.015)	(0.015)	(0.015)	(0.015)
State FEs	N	Y	N	Y
Dependent var.mean	0.361	0.361	0.361	0.361
Observations	388	388	388	388
R-squared	0.231	0.286	0.233	0.286

Note. The sample for this analysis consists of the war-participating cadets from Slave States (i.e., with a slave population higher than 1%). Columns (1)-(2) consider all peers, including those who dropped out. Columns (3)-(4) compare the graduate peers with dropout peers. The standard errors reported in the parentheses are derived from bootstrapping with 400 resampling iterations. $^{***}p < 0.01$, $^{**}p < 0.05$, $^{*}p < 0.1$.

K. Multinomial Logit Results.

Table S14. Peer influence and allegiance choice: Multinomial logit results

Reference group	Join the Confederacy		
	(1)	(2)	(3)
Share Free-State Peers (sd): Join the Union	0.349***	0.336***	0.358***
	(0.109)	(0.109)	(0.123)
Share Free-State Peers (sd): Not in war	0.102	0.063	0.079
	(0.094)	(0.101)	(0.105)
Controls	N	Y	Y
State FEs	N	N	Y
Dependent var. mean	1.212	1.212	1.212
Observations	670	670	670
Pseudo R-squared	0.008	0.181	0.206

Note. The sample for this analysis consists of all cadets from Slave States, including those who did not join either army. The results show that peer influence mainly affected whether to join the Union or Confederacy rather than whether to engage in the war. The control variables consist of *Age in 1860*, *Class Rank*, state level *Slave Population Share (sd)*, and *Cohort*. The standard errors reported in the parentheses are derived from bootstrapping with 400 resampling iterations. ***$p < 0.01$, **$p < 0.05$, *$p < 0.1$.

L. Considering Cash-Crop Employment.

Table S15. Peer composition and allegiance choice: Cash crops

Dependent var	Join the Union: War Participants							
	Slave States				Free States			
	(1)	(2)	(3)	(4)	(5)	(6)	(7)	(8)
Share Free-State Peers (sd)	0.080***	0.058***	0.053***	0.052**	-0.003	-0.009	-0.011	-0.011
	(0.023)	(0.020)	(0.020)	(0.020)	(0.013)	(0.012)	(0.012)	(0.012)
Age in 1860		-0.013	-0.006	-0.006		0.006	0.004	0.003
		(0.015)	(0.015)	(0.016)		(0.007)	(0.007)	(0.007)
Class Rank		0.001	0.001	0.001		-0.001*	-0.001*	-0.001*
		(0.001)	(0.001)	(0.001)		(0.000)	(0.000)	(0.000)
Slave Pop. Share (sd)		-0.203***				-0.004		
		(0.019)				(0.014)		
Cash Crop Employment		-0.259***	-0.234***	-0.230***		-0.627***	-0.638***	-0.621
		(0.056)	(0.054)	(0.054)		(0.187)	(0.187)	(3.384)
Cash Crop $\times$ Share Free-State Peers (sd)				0.027				0.033
				(0.066)				(4.332)
Cohort		-0.014	-0.007	-0.007		0.007	0.006	0.005
		(0.015)	(0.015)	(0.015)		(0.007)	(0.007)	(0.007)
State FEs	N	N	Y	Y	N	N	Y	Y
Dependent var. mean	0.361	0.361	0.361	0.361	0.919	0.919	0.919	0.919
Observations	388	388	388	388	540	540	540	540
R-squared	0.028	0.250	0.300	0.300	0.000	0.084	0.098	0.099

Note. This table presents the impact of the fraction of peers from Free States in a cadet's cohort on that cadet's decision to join the Union. Following White (2024), we introduce a variable *Cash crops*, defined as a dummy equal to 1 if the cadet had a history of employment in cash-crop agriculture. This includes cases where the graduate was recorded as having spent time as a planter (plantation owner) or held any occupation related to the production, processing, sale, or export of cotton, indigo, rice, sugar, or tobacco. Columns (1)-(4) focus on cadets from Slave States and Columns (5)-(8) on those from Free States. The standard errors presented in the parentheses are obtained through bootstrapping with 400 resampling iterations. $***p < 0.01$, $**p < 0.05$, $*p < 0.1$.

M. Considering Appointment State Peers.

Table S16. Peer composition and allegiance choice: Home state and appointed state

Dependent var	Join the Union: War Participants					
	Slave States			Free States		
	(1)	(2)	(3)	(4)	(5)	(6)
Share Free-State Peers (sd)	0.066***	0.052***	0.046**	-0.009	-0.012	-0.014
Appointed State	(0.024)	(0.020)	(0.020)	(0.013)	(0.013)	(0.013)
Controls	N	Y	Y	N	Y	Y
State FEs	N	N	Y	N	N	Y
Dependent var. mean	0.361	0.361	0.361	0.919	0.919	0.919
Observations	388	388	388	540	540	540
R-squared	0.019	0.231	0.284	0.001	0.018	0.032

Note. This table presents the impact of the fraction of peers from Free States in a cadet's cohort on that cadet's decision to join the Union. *Share Free-State peers (sd) of appointed state* is the proportion of free companions calculated based on the place of appointment of graduates. The control variables consist of *Age in 1860*, *Class Rank*, state level *Slave Population Share (sd)*, and *Cohort*. Columns (1)-(3) focus on cadets from Slave States and Columns (4)-(6) on those from Free States. The standard errors presented in the parentheses are obtained through bootstrapping with 400 resampling iterations. ***$p < 0.01$, **$p < 0.05$, *$p < 0.1$.

N. Career Outcomes.

Table S17. Subsequent outcomes in 1865

	OLS			IV		
	Rank	General	Died	Rank	General	Died
	(1)	(2)	(3)	(4)	(5)	(6)
A. Slave-State Cadets (>1%)						
Joining the Union	-1.567***	-0.352***	-0.166***	-1.858***	-0.365***	-0.135*
	(0.194)	(0.054)	(0.046)	(0.346)	(0.099)	(0.071)
Controls	Y	Y	Y	Y	Y	Y
State FEs	Y	Y	Y	Y	Y	Y
Dependent var. mean	6.987	0.438	0.206	6.987	0.438	0.206
Observations	381	381	388	381	381	388
F-statistic				9.911	9.911	10.255
R-squared	0.317	0.190	0.097	0.267	0.149	0.058
B. Slave-State Cadets (1%-33%)						
Joining the Union	-1.505***	-0.314***	-0.170***	-1.717***	-0.365**	-0.168*
	(0.223)	(0.061)	(0.048)	(0.513)	(0.147)	(0.091)
Controls	Y	Y	Y	Y	Y	Y
State FEs	Y	Y	Y	Y	Y	Y
Dependent var. mean	6.802	0.407	0.184	6.802	0.407	0.184
Observations	263	263	266	263	263	266
F-statistic				8.916	8.916	9.189
R-squared	0.314	0.191	0.082	0.262	0.144	0.070

Note. The sample for Panel A consists of the war-participating cadets from Slave States (i.e., with a slave population higher than 1%), and the sample for Panel B excludes those from the High-Slave States who were not significantly influenced by peers. The control variables consist of *Age in 1860*, *Class Rank*, and *Cohort*. Columns (1)-(3) use OLS estimation, while Columns (4)–(6) present IV estimates using birthplace indicators and the share of peers from Free States as instruments for Union affiliation. Columns (1) and (4) feature the military rank as the dependent variable, classified into 11 levels, with General assigned 11 and Third Lieutenant assigned 1. General and Died are binary variables. The rank information on seven cadets is unknown. The standard errors reported in the tables are derived from bootstrapping with 400 resampling iterations. ***$p < 0.01$, **$p < 0.05$, *$p < 0.1$.

O. Correlation between Academic Rank and Military Rank in 1865.

Table S18. Academic rank and military rank in 1865 (The Confederacy vs. The Union)

Dependent var	Rank in 1865: War Participants					
	The Confederacy			The Union		
	(1)	(2)	(3)	(4)	(5)	(6)
Class Rank	0.000	-0.001	-0.001	0.007**	0.007***	0.006**
	(0.003)	(0.003)	(0.003)	(0.003)	(0.003)	(0.003)
Age in 1860		-0.008	-0.027		-0.031	-0.037
		(0.052)	(0.059)		(0.042)	(0.046)
Slave Pop. Share (sd)		0.157*			0.033	
		(0.089)			(0.064)	
Cohort		-0.053	-0.074		-0.085**	-0.092**
		(0.050)	(0.057)		(0.040)	(0.044)
State FEs	N	N	Y	N	N	Y
Dependent var.mean	7.516	7.516	7.516	5.851	5.851	5.851
Observations	281	281	281	636	636	636
R-squared	0.000	0.086	0.163	0.010	0.111	0.129

Note. The sample for this analysis consists of individuals who served in the Confederate Army (Columns (1)-(3)) and those who served in the Union Army (Columns (4)-(6)). This table shows that the association between academic rank and military rank is significant in the Union but not in the Confederacy. The standard errors reported in the parentheses are derived from bootstrapping with 400 resampling iterations. ***$p < 0.01$, **$p < 0.05$, *$p < 0.1$.

References

1. GW Cullum, *Biographical Register of the Officers and Graduates of the US Military Academy at West Point, NY: From Its Establishment, in 1802, to 1890, with the Early History of the United States Military Academy.* (Houghton, Mifflin), (1891).
2. RW Fogel, *Without Consent or Contract: The Rise and Fall of American Slavery.* (WW Norton & Company), (1994).
